# Are New Gender-Neutral Pronouns Difficult to Process in Reading? The Case of *Hen* in SWEDISH

**DOI:** 10.3389/fpsyg.2020.574356

**Published:** 2020-11-10

**Authors:** Hellen P. Vergoossen, Philip Pärnamets, Emma A. Renström, Marie Gustafsson Sendén

**Affiliations:** ^1^Department of Psychology, Faculty of Social Sciences, Stockholm University, Stockholm, Sweden; ^2^Department of Clinical Neuroscience, Karolinska Institutet, Solna, Sweden; ^3^Department of Psychology, New York University, New York, NY, United States; ^4^Department of Psychology, University of Gothenburg, Gothenburg, Sweden

**Keywords:** gender-fair language, gender-neutral pronouns, hen, linguistic change, pronouns, eye-tracking

## Abstract

*Hen* is a Swedish gender-neutral pronoun used for non-binary individuals and as a generic singular pronoun form. Hen was added to the Swedish Academy Glossary (SAOL) in 2015, and opponents of hen have argued that gender-neutral pronouns are difficult to process, and therefore should not be used. As of yet, this has not been empirically tested. This pre-registered study used eye-tracking to experimentally test if hen has a processing cost by measuring the process of understanding whom a pronoun refers to (i.e., pronoun resolution). Participants (*N* = 120) read 48 sentence pairs where the first sentence included a noun referring to a person (e.g., sister, hairdresser, person) and the second included a pronoun referring to the noun. The pronouns were either gendered (she and he) or gender-neutral (hen). The nouns were either neutral (e.g., person, colleague) or gendered, either by lexically referring to gender (e.g., sister, king), or by being associated with stereotypes based on occupational gender segregation (e.g., occupational titles like hairdresser, carpenter). We tested if hen had a greater processing cost than gendered pronouns, and whether the type of noun moderated this effect. The hypotheses were that hen referring to neutral nouns would lead to a smaller processing cost than hen referring to gendered nouns. Furthermore, we hypothesized that hen referring to lexically gendered nouns would lead to larger processing costs than stereotypically gendered role nouns. The processing cost of hen was measured by reading time spent on three regions of the sentence pairs; the pronoun, the spillover region (i.e., the words following the pronoun), and the noun. The only processing cost for hen occurred in the spillover region. The processing cost in this region was greater when hen referred to neutral nouns than when hen referred to a noun associated with gender. In contrast to the hypothesis, the type of gender information associated with the noun did not interact with these effects (i.e., the same reading time for hen following e.g., the queen or carpenter). Altogether, the results do not support that gender-neutral pronouns should be avoided because they are difficult to process.

## Introduction

Language constrains how individuals can be referred to using pronouns. In many languages, only female and male (*binary*) gendered third-person pronouns are available to refer to a single individual (*she* and *he*) (Prewitt-Freilino et al., 2012). When communicators want to refer to a social target of unknown gender, they must choose a pronoun based on their world knowledge and stereotypes (Paterson, 2011). For example, if most hairdressers are observed to be women, *she* is more often chosen to refer to a hairdresser of unknown gender. The importance of stereotype information for the selection of pronouns have been shown in language production studies (e.g., Hellinger and Bußmann, 2001; Gustafsson Sendén et al., 2017). This implies that although communicators know that not *all* hairdressers are women, they may use their gender stereotypes to select a pronoun when referring to a hairdresser (Paterson, 2011). Furthermore, referring to hairdressers as *she* strengthens the association between hairdressers and women (Hellinger and Bußmann, 2001), meaning that gender stereotypes are reinforced through binary pronoun use (Paterson, 2011).

One way to reduce gender stereotyping in communication, is by promoting gender-fair language such as gender-neutral pronouns (Sczesny et al., 2016). In Swedish, the gender-neutral pronoun *hen* was recently introduced. Hen was first promoted by LGBT + communities in early 2010 as a third pronoun along she (hon) and he (*han*) referring to non-binary gender identities. Hen was also promoted by feminist movements as a generic pronoun that aimed to replace the paired pronoun form he/she. The public debate about hen sparked off in 2012 when a children’s book using only hen as a pronoun was published together with a debate article promoting hen. In 2015, hen was added to the official Swedish dictionary (Fahl, 2014). Gender-fair language reforms have historically faced opposition (Blaubergs, 1980; Parks and Roberton, 1998). Opponents have criticized gender-neutral pronouns for being difficult to process (Speyer and Schleef, 2018), and being distracting in communication (Vergoossen et al., 2020). However, whether novel gender-neutral pronouns in fact are more distracting and difficult to process has not been tested experimentally. If they are difficult to process, it is important to know whether difficulties arise because gender-neutral pronouns are new to language users, or because they do not match the gender information associated with the word they refer to Sanford (1985). Gender-neutral pronouns should be more difficult to process when referring to role nouns associated with stereotypes based on occupational gender segregation (e.g., occupational titles like hairdresser and engineer) or lexical gender information (e.g., queen, father), than when referring to neutral role nouns (e.g., person, teacher). So far, studies have tested pronoun resolution in the English language, with singular *they* referring to non-referential nouns (e.g., nurse, runner) and indefinite nouns (e.g., anybody, everyone). *They* was processed faster than generic *he* or *she*, especially when it referred to an indefinite noun (Foertsch and Gernsbacher, 1997; Speyer and Schleef, 2018). It remains to be tested whether novel gender-neutral pronouns, such as *ze* in English and *hen* in Swedish have a greater processing cost than gendered pronouns. The present study employs eye-tracking to investigate the process of pronoun resolution, or the process of understanding whom a pronoun refers to, of gender-neutral pronouns.

If hen is distracting or more difficult to understand than binary pronouns, a greater processing cost should be observed when it is encountered. In written language, a greater processing cost in pronoun resolution is characterized by a longer reading time. Readers spend more time looking at the pronoun and the words following the pronoun, and may revisit the word the pronoun refers to because the reader needs to remind themselves of whom the pronoun referred to. In pronoun resolution, this processing cost may indicate that the encountered gender information is unexpected and that gender information is being updated in the reader’s mental model (Kreiner et al., 2008). It can also indicate that the pronoun is novel to the reader (Gabriel et al., 2018). In the present study, a processing cost is operationalized as the time it takes to read the pronoun, the three words following the pronoun, and the word hen refers to.

Past research has investigated gender stereotype activation in pronoun resolution (e.g., Kennison and Trofe, 2003; Kreiner et al., 2008; Khan and Daneman, 2011; Cunnings et al., 2014) by combining role nouns such as hairdresser and king with congruently and incongruently gendered binary pronouns. In these studies, participants read two-clause sentences. In the first clause, a role noun was introduced, and in the second clause a reflexive pronoun referred to the role noun. For example, “Yesterday the king left London after reminding himself [herself] about the letter” (Kreiner et al., 2008, p. 258). A mismatch between the gender information of noun and the pronoun (“the king… herself”), increased the reading time compared to a match between the gender associated with the noun and pronoun (“the king… himself”). The authors suggest that this processing cost arises because the reader needs to revise the gender association evoked by the noun (Kreiner et al., 2008). This supports the finding that pronouns matching antecedents in terms of gender information are read faster than other combinations (Sanford, 1985). In research on gender-neutral pronouns, Foertsch and Gernsbacher (1997) found that the processing cost for singular *they* was lowest when it referred to an indefinite noun (e.g., “everyone”). In the present study, we expect that a new gender-neutral pronoun (hen) that refers to a noun indicating masculine or feminine (binary) gender, will lead to a greater processing cost in comparison to a binary pronoun that matches the noun’s gender (Hypothesis 1). We also expect that the processing cost of hen will be smaller when hen refers to a noun of neutral gender (e.g., “person”) compared to when hen refers to a noun associated with gender (e.g., king) (Hypothesis 2).

In the present study, two types of nouns are used. Role nouns that lexically refer to gender (e.g., king, father), or occupational titles associated with gendered occupational segregation (e.g., secretary, engineer). In English, the role noun “secretary” does not lexically refer to gender but can still evoke the reader’s mental representation of a woman because secretaries are predominantly women. Because hen can be used both as a generic and a non-binary pronoun, using hen in reference to lexically gendered nouns should activate other gender representations than when hen refers to stereotypical gender nouns. For example, hen referring to “the librarian” could indicate that the gender of the librarian is not known rather than the librarian being non-binary, whereas referring to “the queen” as hen indicates that the queen has a non-binary gender identity. The generic use of hen as an alternative for the paired form *han/hon*[he/she] is also more accepted than using hen for non-binary individuals, or individuals whose gender information is thought to be known (Bradley, 2020; Vergoossen et al., 2020). Therefore, we expect the largest processing cost when hen refers to lexically gendered nouns compared to role nouns that are associated with gender through gender stereotypes (Hypothesis 3). Because difficulties in processing could be a result of a new word, we control for participants’ previous experience with hen in all hypothesis testing.

## Materials and Methods

Method, hypotheses, and analyses were preregistered before data collection was completed^1^. Analyses that differed from the pre-registration are described below.

### Participants

We targeted a sample size of 120 individuals based on simulations of effects observed from a pilot data set (*N* = 19). Altogether, 130 students (73% women, M_age_ 25.8, *SD* = 6.5) completed the reading task. Participants (*n* = 10) who moved too much during the experiment were not included in data analysis. All participants reported they were fluent in Swedish, and reported no reading difficulties (e.g., dyslexia) or sight-related problems (e.g., nystagmus). Participation was rewarded with course credit or a movie ticket with a value of approximately €10. Participants reported “yes” to the question whether they were familiar with hen, and on average they had a positive attitude toward hen (*M* = 3.30 on a 4-point scale).

### Design and Materials

The design was a 2 (role noun: lexical/stereotypical gender) × 2 (noun gender: gendered/neutral) × 2 (pronoun gender: congruent/hen) within-participant conditions with three processing measures as the outcome variables. The experiment included 48 experimental sentence pairs. All sentence pairs had the same structure, such that the first sentence began with a role noun and the second sentence with a pronoun (see the Supplementary Material for the full list of sentence pairs).

Table 1 presents all combinations of nouns and pronouns. As can be seen, half of the 48 sentences included a role noun associated with lexical gender, and half included a role noun associated with stereotypical gender. Within the role noun categories, 12 nouns were binary gendered (6 feminine, 6 masculine), and 12 were neutral. The pronouns were either binary and congruent with the gender associated with the noun (hon[she] or han[he]) or hen. The semantic content of the sentences was kept neutral to avoid gender stereotyping based on other information than the role nouns and pronouns. For example:

**TABLE 1:** Overview of combinations of nouns and pronouns in the stimulus materials.

**Noun category**	**Noun gender**	**Pronoun**
Lexical gender	Gendered	She or he congruent with noun
		Hen
	Neutral	She or he
		Hen
Stereotypical gender	Gendered	She or he congruent with noun
		Hen
	Neutral	She or he
		Hen

The woman[man][person] signed the contract.
Hon[Han][Hen] looked forward to starting the new job.

The nurse[carpenter][architect] signed the contract.
Hon[Han][Hen] looked forward to starting the new job.

The stereotypical role nouns were selected from Swedish labor statistics (Statistics Sweden, 2014) and pilot tested to assure that the occupations were perceived as either dominated by women or men, or balanced. In the pilot, participants (*N* = 44) estimated the proportion of women and men in 89 occupations. The 6 occupations that were perceived as most women-dominated (mean 82.44% women, *SD* = 7.41), men-dominated (mean 82.87% men, *SD* = 6.12) or balanced (mean 48.88% women, *SD* = 3.55) were included in the stimulus material (see Supplementary Material for the full list of included role nouns and their associated ratings). The lexically gendered role nouns were selected with the criterion that they should have a neutral, feminine and masculine form available with similar semantic content, for example the “parent,” “mother,” “father,” or “sibling,” “sister,” “brother.”

The reading task also included 40 filler sentence pairs, of similar length and neutral content as the target sentence pair (e.g., “Kim has borrowed a book from the library. The book is about life in medieval Sweden”). All sentence pairs were presented in a randomized order. Comprehension questions were presented together with 50% of the sentences to assure that participants stayed focused on the reading task and to measure if the type of pronoun affected reading comprehension). On average, participants answered 93% of all comprehension questions correctly (*SD* = 6.3%). There was no effect of pronoun on the performance on the comprehension questions (Bayesian multilevel regression; *b* = −0.03, *SE* = 0.16, *CrI* (95% Credible Interval) = [−0.34, 0.29], *BF*_10_ (Bayes Factor) = 0.31).

### Apparatus

An eye-tracker (SMI IView X Hi-Speed eye-tracker with a sampling rate of 1250 Hz) was used to record eye movements. The sentence pairs were presented using the PsychoPy software (Peirce et al., 2019). The font used was Courier New, 30 pt and the screen was approximately 70 cm from the participants’ eyes. Participants viewing was binocular, but eye-tracking data was collected only for the dominant eye. Before and halfway through each experiment there was a calibration procedure to estimate the visual degree of error. If the average deviation of the gaze position exceeded 0.5°, the calibration was redone (as recommended by Holmqvist et al., 2011). Calibration was successful for all participants (average calibration accuracy = 0.41°, *SD* = 0.08).

### Procedure

The experiment was presented as a memory study, where participants were instructed to read and answer questions at their own pace. The study was carried out in accordance with the national guidelines on ethical research established by the Swedish Research Council^2^. All participants gave their informed consent before participating in the survey.

Participants were blind to the study’s purpose. The participants took place at the eye-tracker, where a chin support and forehead rest supported the participant’s head, and were told to avoid any body movements. After the calibration, they completed three practice trials to familiarize themselves with the experimental procedure. After the reading task, participants responded to questions including demographic information and about their previous experience with *hen*.

### Analyses

#### Data Preparation

**FIGURE 1:**
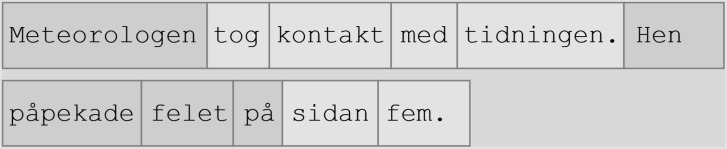
Regions of interest in stimulus sentence pairs. “The meteorologist contacted the newspaper. Hen pointed out the error on page five.” Note that the location of the pronoun varied between stimuli.

Eye-tracking data for the three word regions were collected with the BeGaze software (SensoMotoric Instruments, 2014). The processing cost was measured for the pronoun, the three words following the pronoun (*pronoun spillover region*), and the noun. Figure 1 shows an example of how each region was defined before extracting data. The height of the region (in pixels) is the approximate line height of the row, and the width of the region (in pixels) includes the target word(s) and half of the space between the region and the words preceding and following the word. For words located at the end of the line, an additional centimeter was added to the right of the word region. This was done to capture fixations to the right of a pronoun observed in pilot data when the pronoun was located at the end of a sentence (see Figure 1). Because of this, we included the size of the word region (in pixels) instead of reading time per character. The visual location of the pronoun varied between stimuli due to variations in sentence length. As the pronoun length was identical between the conditions (hen compared to hon and han), the defined pronoun region sizes were kept identical between pronouns.

Based on the pilot data (*N* = 19), we selected a measurement of reading time for each of the regions (as defined in SensoMotoric Instruments, 2014). For the pronoun region, we measured the first-pass fixation duration, which is the duration of the very first fixation on this region. For the spillover region, we measured the dwell time duration, which is the sum of all fixations and saccades within this region. For the noun region, we measured duration, which is defined as the sum of all fixation durations on this region after exiting the noun region for the first time.

The variable “Experience with hen” was based on the question “How often to you encounter hen in your everyday life?”, for which the response alternatives were “nearly every day” (1), “at least once a week” (2), “a few times a month” (3), “more rarely” (4) or “never” (5). We combined participants responding response alternative 1, 2 or 3 into the “experienced with hen” group, and response 4 and 5 into a “little experience with hen” group for our analyses.

Some data was removed prior to analysis, following our pre-registered analysis plan. Individual trials were excluded from the analyses if the head movement was observed by the experiment leader (14.5% of total trials) or a trial’s total reading time deviated more than two standard deviations from the mean (3.7% of total trials). In total, 1915 trials (18.1%) were removed. All participants answered more than 80% of the comprehension questions correctly.

### Confirmatory Analyses

The preregistered analysis plan (Vergoossen et al., 2018) guided the testing of Hypotheses 1–3, using Bayesian mixed-effect models that allowed all intercepts and slopes vary both by subject and stimulus ID (Judd et al., 2012; McElreath, 2015). The data were analyzed in R (R Core Team, 2017) using the R package brms (Bürkner, 2017) in the Stan computational framework^3^. The models controlled for light levels (which were kept constant in the experiment room), previous experience with *hen*, the size of the reading region and trial number as nuisance regressors. All variables were centered before analysis, to allow for easier interpretation of the estimates and for identification of main effects and interactions. To improve convergence and avoid overfitting, we specified mildly informative, zero-centered, conservative priors and preregistered these. To assess the confirmatory effects, Bayes Factors (BF) were computed using the Savage-Dickey method. A reported BF_10_ will indicate how many times more likely it is to observe the data under H1 compared to H0. Conversely, a reported BF_01_ will indicate how many times more likely it is to observe the data under H0, compared to H1. A Bayes Factor of at least 10 will be the criterium for strong evidence (Wagenmakers et al., 2010). Regression coefficients and their 95% credible intervals are reported for all results to further aid assessment of effects.

A few adjustments were made to the preregistered analysis plan. For the spillover region model, we used a hurdle lognormal distribution instead of a Gaussian distribution, because the data for these regions were heavily skewed (skewness = 1.36) instead of normal, and zero-inflated (see the Supplementary Material for a comparison between using a model with a Gaussian distribution and a hurdle lognormal distribution). In these data we observed that some participants did not fixate the pronoun region or refixate the noun region at all. These non-fixations were entered as zero into the model.

## Results

The processing cost of hen was measured in three regions: (a) pronoun, (b) spillover region, and (c) noun. All participants reported being familiar with hen. One-third (33.73%) of the participants reported encountering hen in their everyday life a few times a month or more, while the rest of the participants (66.27%) reported rarely ever or never encountering hen.

### Processing Costs of Hen Compared to Gendered Pronouns

**FIGURE 2:**
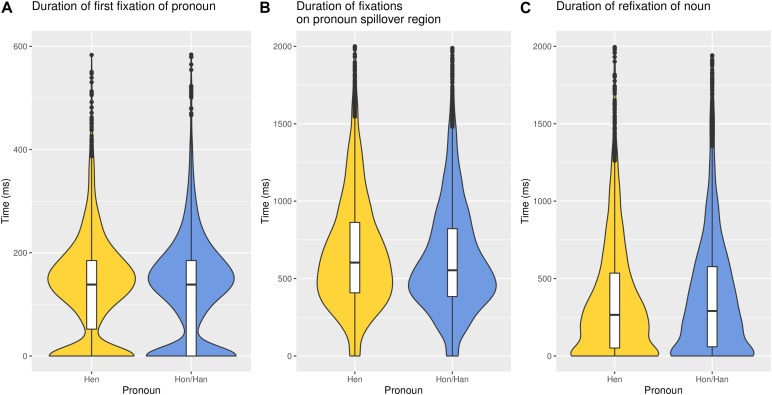
Reading time (ms) for hen and hon/han in each of the three regions: **(A)** pronoun, **(B)** spillover region, and **(C)** noun. The graph has been truncated.

Hypothesis 1 stated that hen should lead to a greater processing cost than a gendered pronoun. A robust effect of pronoun was found for the pronoun spillover region (*b* = 0.04, *SE* = 0.01, *CrI* = [0.02, 0.05], *BF*_10_ = 10). Participants’ reading time for the spillover region was 43 ms longer after encountering *hen* compared to she or he. The average reading time for the stimuli was 5018 ms, meaning that hen led to a processing cost of 0.86%. There was no greater processing cost for the first fixation on the pronoun or refixation of the noun region for hen compared to she or he (*b* = 0.005, *SE* = 0.01, *CrI* = [−0.01, 0.03], *BF*_10_ = 0.12; *b* = −0.003, *SE* = 0.02, *CrI* = [−0.04, 0.03], *BF*_10_ = 0.20, respectively), as shown in Figure 2. The complete models including all variables are presented in the Supplementary Material.

### Processing Costs of Hen Referring to a Neutral vs. a Gendered Noun

**FIGURE 3:**
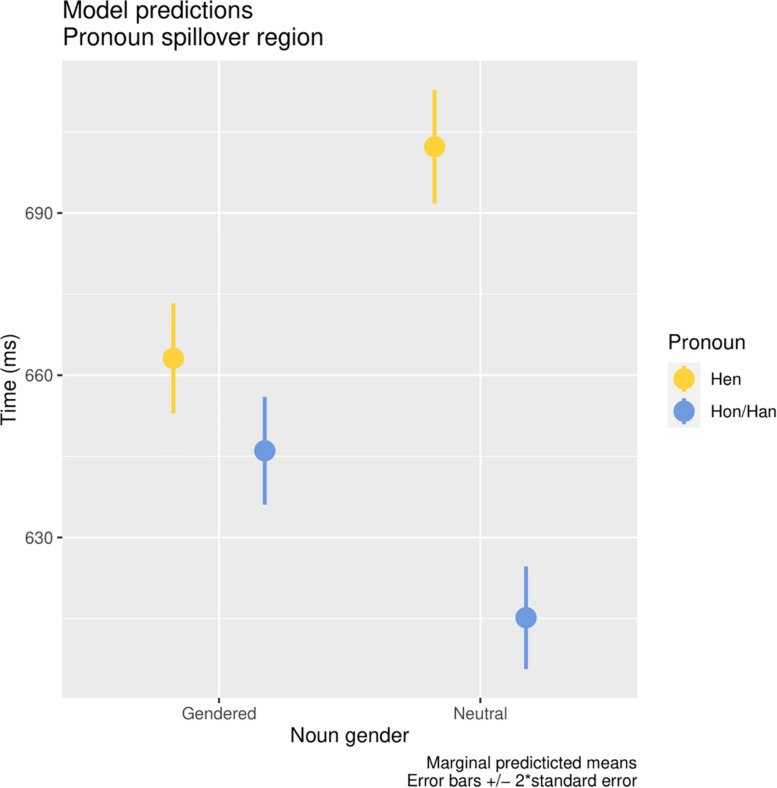
The marginal predicted means for the interaction between pronoun (neutral or gendered) and the type of noun (neutral or gendered) in the pronoun spillover region. The graph has been truncated.

**FIGURE 4:**
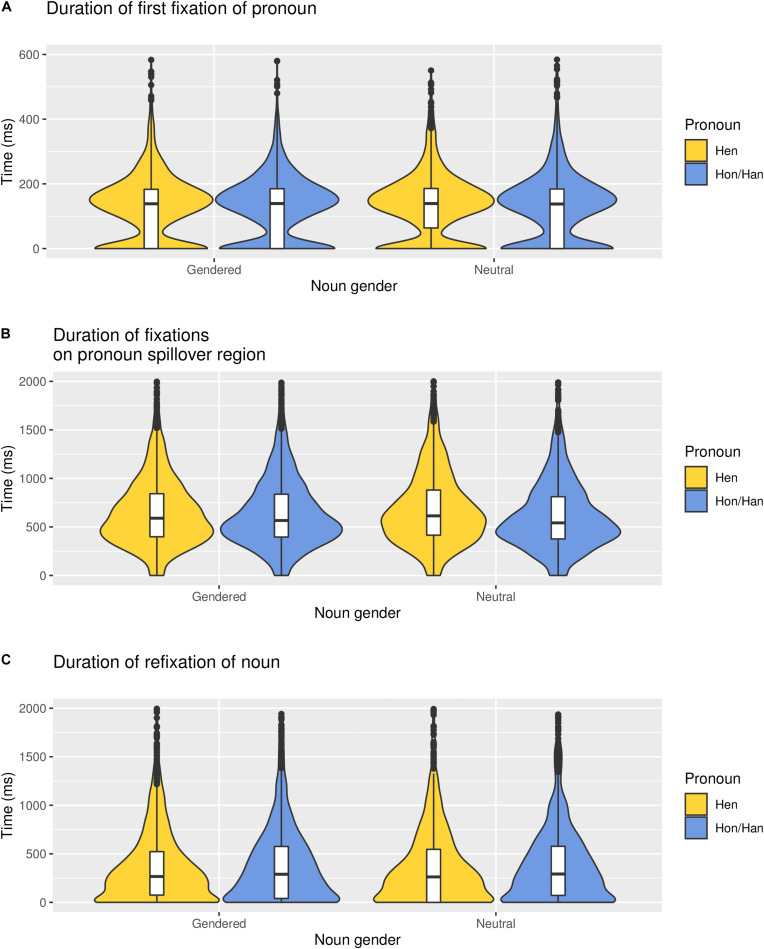
Reading time (ms) for hen and hon/han referring to gendered and neutral nouns in each of the three regions: **(A)** pronoun, **(B)** spillover region, and **(C)** noun. The graph has been truncated.

Hypothesis 2 stated that a neutral role noun in the first sentence should decrease the processing cost of *hen*, compared to when *hen* followed a role noun associated with binary gender. The Bayes Factor indicated evidence for the interaction between pronoun and gender of the role noun in the pronoun spillover region: *b* = −0.03, *SE* = 0.006, *CrI* = [−0.04, −0.01], *BF*_10_ = 25.04. However, this effect was in the opposite direction than hypothesized; reading hen referring to a neutral noun leads to a greater processing cost (720 ms, *SD* = 430 ms) in comparison to hen referring to a binary gendered role noun (680 ms, *SD* = 400 ms) (see Figure 3). For the other regions, strong evidence for no interaction appeared: pronoun: *b* < 0.001, *SE* = 0.007, *CrI* = [−0.01, 0.01], *BF*_01_ = 14.29; noun: *b* = −0.008, *SE* = 0.01, *CrI* = [−0.03, 0.02], *BF*_01_ = 6.25 (see Figure 4).

### Processing Hen Referring to Lexically Gendered Nouns vs. Stereotypically Gendered Nouns

**FIGURE 5:**
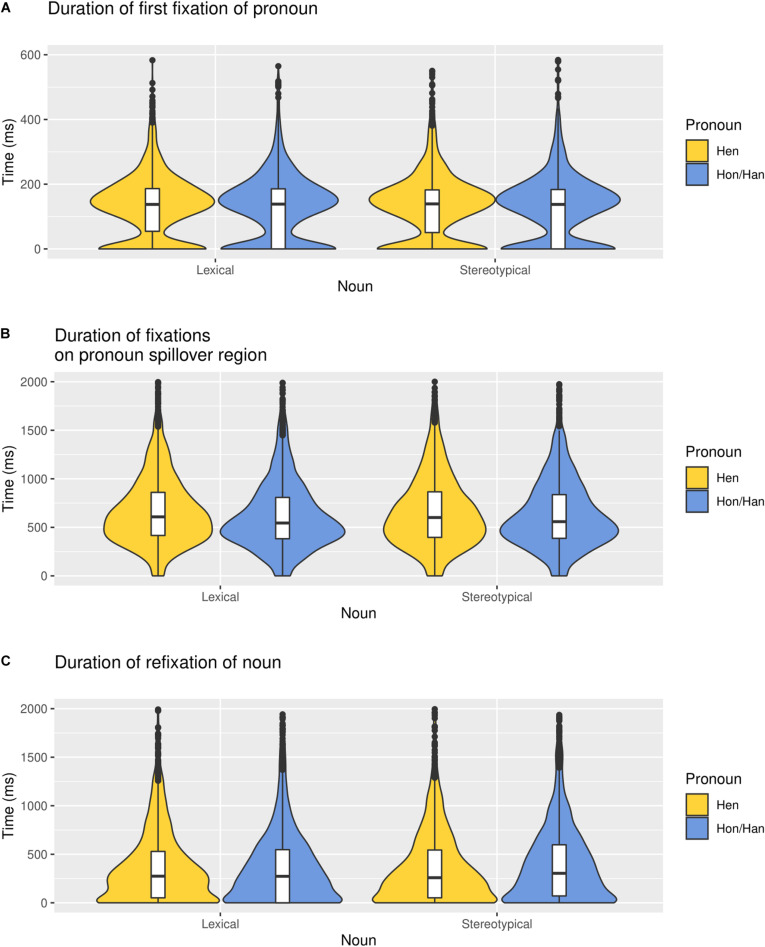
Reading time (ms) for hen and hon/han referring to lexically gendered nouns and stereotypically gendered nouns in each of the three regions: **(A)** pronoun, **(B)** spillover region, and **(C)** noun. The graph has been truncated.

Hypothesis 3 stated that hen referring to a noun lexically denoting gender should lead to a greater processing cost than hen referring to nouns stereotypically associated with gender. Strong evidence for a lack of an interaction appeared between pronoun and the type of gender information associated with the noun for any region (pronoun: *b* < 0.001, *SE* = 0.007, *CrI* = [−0.01, 0.01], *BF*_01_ = 16.67; pronoun spillover: *b* = 0.02, *SE* = 0.008, *CrI* = [−0.001, 0.03], *BF*_01_ = 33.33; noun: (*b* = 0.01, *SE* = 0.01, *CrI* = [−0.01, 0.04], *BF*_01_ = 5.26), as shown in Figure 5. Thus, there was no evidence for a difference in processing cost for nouns containing definitional gender information (e.g., queen) compared to stereotypical gender information (e.g., carpenter).

### Effect of Participant’s Experience With *Hen* on Processing *Hen*

Self-reported previous experience with hen could affect how hen is processed. No interaction was found between self-reported previous experience with hen and type of pronoun for any of the three regions (pronoun: *b* = −0.004, *SE* = 0.02, *CrI* = [−0.03, 0.03], *BF*_10_ = 0.16; pronoun spillover: *b* < 0.001, *SE* = 0.02, *CrI* = [−0.03, 0.03], *BF*_10_ = 0.08; noun: (*b* = −0.01, *SE* = 0.03, *CrI* = [−0.07, 0.05], *BF*_10_ = 0.31), indicating that previous experience with hen did not explain the main effect of pronoun. Exploratively, we tested whether there was an effect of trial on the processing cost of hen, where we expected to find a smaller processing cost toward the end of the experiment after repeated exposure to hen in the stimulus material. We found a robust effect of trial on the pronoun spillover region (*b* = −0.08, *SE* = 0.02, *CrI* = [−0.12, −0.05], *BF*_10_ = 1130), where there was less time spent reading the more trials had been completed. However, there was no difference between hen and the gendered pronoun condition (*b* = −0.02, *SE* = 0.01, *CrI* = [−0.04, 0.01], *BF*_10_ = 0.14).

### Effect of Personal Attitudes on Processing *Hen*

Exploratively, we tested whether personal attitudes affected the processing cost associated with hen. Strongly identifying as a feminist was related to faster reading behavior in all three regions of interest regions (pronoun: *b* = −0.06, *SE* = 0.02, *CrI* = [−0.10, −0.03], *BF*_10_ = 210; pronoun spillover: *b* = −0.10, *SE* = 0.03, *CrI* = [−0.15, −0.05], *BF*_10_ = 170; noun: *b* = −0.10, *SE* = 0.03, *CrI* = [−0.16, 0.03], *BF*_10_ = 15.40). However, identifying as a feminist did not interact with the type of pronoun (pronoun: *b* = 0.002, *SE* = 0.008, *CrI* = [−0.01, 0.02], *BF*_10_ = 0.08; pronoun spillover: *b* = −0.01, *SE* = 0.009, *CrI* = [−0.03, 0.01], *BF*_10_ = 0.09; region: (*b* = 0.01, *SE* = 0.02, *CrI* = [−0.02, 0.04], *BF*_10_ = 0.20).

Similarly, a negative attitude toward hen was not associated with a greater processing cost for hen (see Supplementary Material).

## Discussion

This eye-tracking experiment tested whether there was a processing cost associated with reading the new gender-neutral pronoun hen in Swedish during pronoun resolution. Overall, on a wide range of measures predicted by the existing literature on reading and pronoun use (e.g., Carreiras et al., 1996; Kennison and Trofe, 2003; Esaulova et al., 2013; Reali et al., 2015) we largely found no effects of hen on reading, and strong evidence for null effects. The only exception to this was a small processing cost in the pronoun spillover region, indicating a slowing in reading after encountering hen. This cost was unrelated to the type of gender information in the noun or previous experience with hen. Furthermore, we found no evidence of any effect on comprehension resulting from hen.

The processing cost of hen was only found in the pronoun spillover region. Participants did not fixate hen longer than gendered pronouns, nor did they spend more time refixating the noun in the first sentence. This is in line with the findings from studies on singular they, where they did not have a greater processing cost than gendered pronouns (Foertsch and Gernsbacher, 1997; Speyer and Schleef, 2018). The small processing cost that was found for hen could be due to hen being a new gender-neutral pronoun, whereas they is a previously existing word in English. However, novelty, as measured by self-rated previous experience to hen, did not affect the processing cost of hen, nor did the exposure to hen within the experiment as reflected by the interaction between amount of completed trials and type of pronoun.

The processing cost found for the spillover region was in line with past studies finding a processing cost of a mismatch between noun and pronoun gender. However, these studies found a processing cost of greater magnitude, often also comprising a longer fixation of the pronoun and refixations of the noun (e.g., Carreiras et al., 1996; Cacciari et al., 1997; Kennison and Trofe, 2003; Kreiner et al., 2008). In these studies, the processing cost for a gender mismatch between noun and pronoun was thought to be due to a revision of gender information in the mental model that is created of the situation (Carreiras et al., 1996). If our finding of a processing cost would be due to a mismatch between the genderedness of the noun and the gender-neutral pronoun, we would expect no processing cost when hen refers to a gender-neutral noun. As in the present study evidence for the opposite was found, namely a small processing cost for hen referring to a neutral noun, this will require further investigation.

It is also possible that hen, which can be used generically to refer to people of any gender, does not lead to a gender incongruency effect like an incongruent she or he does when it is understood as a generic form instead of he or she. Hen can also be used as a pronoun for non-binary individuals. As we do not have data on how participants interpreted hen in the sentences they read, it remains to be investigated whether the different interpretations of the pronoun affect pronoun resolution differently. It also remains to be investigated if hen affects whether the sentence is perceived as containing an error (i.e., containing an incorrect pronoun) and whether this leads to readers not achieving a coherent interpretation of the sentence.

Exploratively, we investigated the effect of personal factors and attitudes on the processing cost of hen. We did not find evidence for gender or age affecting the processing cost of hen. We expected personal attitudes, such as strongly identifying as a feminist, to affect the processing cost of hen. We unexpectedly found evidence for personal attitudes such as strongly identifying as a feminist affecting reading behavior, but such personal attitudes did not interact with type of pronoun. However, it is important to note that previous research has focused on comparing gender congruent and incongruent information in the process of pronoun resolution to establish the extraction of gendered information in reading (e.g., Carreiras et al., 1996; Kennison and Trofe, 2003; Esaulova et al., 2013; Reali et al., 2015). Our finding that identifying as a feminist was related to a smaller processing cost in general, for stimuli containing varying levels of gendered information, could indicate that personal attitudes affect how gendered information is processed in general. For example, gender information based on stereotypes may affect the gender information derived from occupational titles less in individuals who resist gender stereotypes. The relationship between personal factors and attitudes and their relationship to processing gender in language remains to be investigated further, especially considering the limitations of our sample.

A limitation of the present study is that the sample was homogenous in terms of age, education level and in their predominantly positive opinion of hen. There were few participants with a negative opinion of hen to compare whether the processing cost of hen is also affected by the emotions evoked by the pronoun. The homogeneity in terms of education level may control for effects of variability in reading skill, which has been found to affect the processing cost when encountering unexpected words in reading (Daneman et al., 2007). We also did not account for the possibility of participants having experience with gender-neutral pronouns in other languages they were proficient in. It remains to be investigated whether the processing cost of hen is affected by a negative opinion of hen, a lower reading skill, and experience with gender-neutral pronouns in other languages than Swedish.

The present study was the first to investigate the process of pronoun resolution for a new gender-neutral pronoun, specifically the Swedish pronoun hen. The pronoun was studied in contexts in which it unambiguously referred to a noun in a short sentence pair (based on e.g., Kennison and Trofe, 2003; Kreiner et al., 2008). The processing of gender-neutral pronouns in a more demanding and ambiguous context remains to be investigated. The context provided in our stimuli was aimed to be neutral in terms of gendered cues, and more research remains to be done on how gender-neutral pronouns are understood in a more naturalistic and gendered context. Lastly, it remains to be investigated whether the processing cost for hen varies depending on whether it is used as a pronoun for non-binary people, or as a generic pronoun form to refer to anyone. There may be an additional cost of not resolving the question of what the protagonist’s gender is.

In sum, our study does not support the notion that new gender-neutral pronouns are distracting or difficult to process. Altogether, our results show that a new gender-neutral pronoun (hen) might be somewhat more difficult to process, but that this effect is small and does not affect reading comprehension. Therefore, the results of this study do not support the claim that hen is confusing or that it is associated with a high processing cost.

## Data Availability Statement

The datasets presented in this study can be found in online repositories. The datasets can be found at the following link: https://su.figshare.com/articles/dataset/Open_data_Are_new_gender-neutral_pronouns_difficult_to_process_in_reading_The_case_of_hen_in_Swedish/13143158, doi: 10.17045/sthlmuni.13143158.

## Ethics Statement

The studies involving human participants were reviewed and approved by the Swedish Research Council. The patients/participants provided their written informed consent to participate in this study.

## Author Contributions

HV pre-registered the study, collected the data, and wrote the first draft of the manuscript. HV and PP performed the statistical analyses. All authors conceptualized the idea, participated in the planning of the studies, interpretation and discussion of results, and in writing the manuscript.

## Conflict of Interest

The authors declare that the research was conducted in the absence of any commercial or financial relationships that could be construed as a potential conflict of interest.

## Footnotes

1 https://osf.io/exkdx/?view_only=928fac25b06946d3bd34aab571c8ea77

2 Retrievable at https://publikationer.se/en/good-research-practice

3 mc-stan.org
