# Supplementary material for: Are New Gender-Neutral Pronouns Difficult to Process in Reading? The Case of Hen in SWEDISH
Source: Front Psychol. 2020 Nov 10;11:574356. doi: 10.3389/fpsyg.2020.574356 (PMC7683789; doi:10.3389/fpsyg.2020.574356)
Supplement: Supplementary file 1 [file Data_Sheet_1.docx]

***Supplementary Material***

# Ratings of occupational titles

Overview of all occupational titles included in the study and how their estimated proportion of women were rated.

## Occupations associated with a high proportion of women

*Swedish title English title Estimated proportion women*

Skönhetsterapeuter Cosmetologists 88.85%

Barnmorskor Midwives 86.08%

Förskolelärare Preschool teacher 70.74%

Vårdbiträden Nurse’s aides 84.32%

Sekreterare Secretaries 77.87%

Sjuksköterskor Nurses 75.53%

## Occupations associated with a high proportion of men

*Swedish title English title Estimated proportion women*

Pizzabagare Pizza bakers 6.47%

Byggnadsarbetare Construction workers 10.21%

Officerare Officers 11.89%

Målare Painters 12.58%

Snickare Carpenters 15.23%

Piloter Pilots 17.66%

## Occupations associated with an equal distribution women/men

*Swedish title English title Estimated proportion women*

Högskolelärare University teachers 56.37%

Författare Authors 54.11%

Konstnärer Artists 53.83%

*Swedish title English title Estimated proportion women*

Skådespelare Actors 52.87%

Sångare Singers 51.84%

Doktorander PhD Students 50.76%

ST-läkare Resident physicians 50.40%

Journalister Journalists 49.79%

Meteorologer Meteorologists 48.78%

Musiker Musicians 47.16%

Marknadsförare Marketers 44.97%

# Stimuli

The original sentences (in Swedish) included in the study:

1. Förskolläraren/Doktoranden/Tjejen/Studenten bjöds in för att hålla en föreläsning. Hen/Hon var glad av att bli tillfrågad.
2. Sjuksköterskan/ST-läkaren/Mamman/Partnern var upprörd och blek. Hen/Hon hade just fått veta att dottern blivit inlagd på sjukhus.
3. Sekreteraren/Journalisten/Kvinnan/Kollegan skrev under dokumenten. Hen/Hon lade dem sedan på postlådan.
4. Vårdbiträdet/Mäklaren/Mostern/Vännen fick en inbjudan till en middag. Hen/Hon tackade nej eftersom det var mycket att göra på jobbet.
5. Barnmorskan/Skådespelaren/Farmodern/Pensionären bäddade sängen med nya påslakan. Hen/Hon lade smutstvätten i tvättkorgen.
6. Skönhetsterapeuten/Musikern/Systern/Kusinen kom ofta sent till jobbet. Hen/Hon hade rykte om sig att vara svår att jobba med.
7. Förskolläraren/Doktoranden/Tjejen/Studenten kom tidigt till ceremonin. Hen/Hon var förvånad över att så många människor redan var på plats.
8. Sjuksköterskan/ST-läkaren/Mamman/Partnern försökte ge en fullständig beskrivning av händelsen. Hen/Hon verkade förvirrad och mindes mycket lite.
9. Sekreteraren/Journalisten/Kvinnan/Kollegan skulle skriva en omfattande rapport. Hen/Hon funderade på att be sin chef om hjälp.
10. Vårdbiträdet/Mäklaren/Mostern/Vännen har tagit tjänstledigt för studier. Hen/Hon är tillbaka på jobbet nästa år.
11. Barnmorskan/Skådespelaren/Farmodern/Pensionären hade jobbat länge i staden. Hen/Hon kände nästan alla som bodde där.
12. Skönhetsterapeuten/Musikern/Systern/Kusinen gillade att gå på zoo med familjen. Hen/Hon tyckte att djurparker var det perfekta stället för utflykter.
13. Förskolläraren/Doktoranden/Tjejen/Studenten läste igenom instruktionerna flera gånger. Hen/Hon ville verkligen göra sitt bästa.
14. Sjuksköterskan/ST-läkaren/Mamman/Partnern tvättade såret försiktigt. Hen/Hon noterade att det hade börjat läka fint.
15. Sekreteraren/Journalisten/Kvinnan/Kollegan berättade om det nya jobbet. Hen/Hon såg fram emot att få nya uppgifter.
16. Vårdbiträdet/Mäklaren/Mostern/Vännen inspekterade rummet i förväg. Hen/Hon såg att allt var i perfekt ordning.
17. Barnmorskan/Skådespelaren/Farmodern/Pensionären gick till stranden varje helg. Hen/Hon tyckte om att vandra i vattenbrynet.
18. Skönhetsterapeuten/Musikern/Systern/Kusinen läste tidningen varje dag. Hen/Hon försökte hålla sig informerad om händelser i världen.
19. Förskolläraren/Doktoranden/Tjejen/Studenten skrev under kontraktet. Hen/Hon såg fram emot att börja på nya jobbet.
20. Sjuksköterskan/ST-läkaren/Mamman/Partnern jobbade utomlands i ett år. Hen/Hon ringde hem till familjen varje kväll.
21. Sekreteraren/Journalisten/Kvinnan/Kollegan tog emot presenten från föreningen. Hen/Hon tackade alla för den trevliga dagen.
22. Vårdbiträdet/Mäklaren/Mostern/Vännen skrev en inbjudan till julfesten. Hen/Hon hade en mycket lättläst handstil.
23. Barnmorskan/Skådespelaren/Farmodern/Pensionären firade sin 80-årsdag. Hen/Hon var tacksam över att ha fått uppleva så mycket.
24. Skönhetsterapeuten/Musikern/Systern/Kusinen bröt benet kort före semestern. Hen/Hon avbokade därför cykelresan.
25. Snickaren/Meteorologen/Killen/Praktikanten skickade ett viktigt e-postmeddelande. Hen/Han fick svar redan inom en timme.
26. Pizzabagaren/Högskoleläraren/Pappan/Föräldern läste blanketten noggrant. Hen/Han noterade att det fanns flera stavfel på första sidan.
27. Piloten/Författaren/Mannen/Medarbetaren var törstig efter föredraget. Hen/Han frågade efter ett glas vatten.
28. Målaren/Marknadsföraren/Morbrodern/Släktingen cyklade flera mil varje dag. Hen/Han gillade att motionera.
29. Officeraren/Sångaren/Farfadern/70-åringen log framför kamerorna. Hen/Han var uppklädd för ceremonin.
30. Byggnadsarbetaren/Konstnären/Brodern/Syskonet fick en tävlingsinbjudan på posten. Hen/Han bestämde sig för att delta och fyllde i formuläret.
31. Snickaren/Meteorologen/Killen/Praktikanten var lättstött och svår att komma överens med. Hen/Han verkade inte vara omtyckt av någon på jobbet.
32. Pizzabagaren/Högskoleläraren/Pappan/Föräldern pratade om sin relation. Hen/Han beskrev dagen då de hade träffats första gången.
33. Piloten/Författaren/Mannen/Medarbetaren hade en kort rast. Hen/Han skulle fortsätta arbeta klockan två.
34. Målaren/Marknadsföraren/Morbrodern/Släktingen tog emot hundrakronorssedeln. Hen/Han anade att det kunde vara en förfalskning.
35. Officeraren/Sångaren/Farfadern/70-åringen jobbade ideellt på ett djurhem. Hen/Han fick mycket positiv uppmärksamhet.
36. Byggnadsarbetaren/Konstnären/Brodern/Syskonet spelade tv-spel flera timmar varje dag. Hen/Han skulle kunna spela ännu mer om det var möjligt.
37. Snickaren/Meteorologen/Killen/Praktikanten brukar sova lite under nätterna. Hen/Han har fått barn nyligen.
38. Pizzabagaren/Högskoleläraren/Pappan/Föräldern brukar hämta barnen från skolan. Hen/Han är alltid lite tidig.
39. Piloten/Författaren/Mannen/Medarbetaren gillade inte alls idén. Hen/Han tyckte att de skulle hitta på någonting annat.
40. Målaren/Marknadsföraren/Morbrodern/Släktingen är en mycket trevlig person. Hen/Han ställer alltid upp när någon behöver hjälp.
41. Officeraren/Sångaren/Farfadern/70-åringen dammsög golvet i vardagsrummet. Hen/Han skulle få besök på kvällen.
42. Byggnadsarbetaren/Konstnären/Brodern/Syskonet ska åka på semester i Spanien. Hen/Han gillar att sola och bada.
43. Snickaren/Meteorologen/Killen/Praktikanten tog kontakt med tidningen. Hen/Han påpekade felet på sidan fem.
44. Pizzabagaren/Högskoleläraren/Pappan/Föräldern letade upp boken online. Hen/Han tänkte köpa den som present till sin kompis.
45. Piloten/Författaren/Mannen/Medarbetaren bar lådan uppför trappan. Hen/Han undrade till vilket rum lådan skulle.
46. Målaren/Marknadsföraren/Morbrodern/Släktingen hade alltid älskat djur. Hen/Han skaffade en hund för ett år sedan.
47. Officeraren/Sångaren/Farfadern/70-åringen gick i pension förra månaden. Hen/Han kommer att resa kommande året.
48. Byggnadsarbetaren/Konstnären/Brodern/Syskonet gillar inte måndagar. Hen/Han har svårt att komma igång efter helgen.

# Comparison using a Gaussian distribution with a hurdle lognormal distribution in a model

**Figure S1**. The posterior predictive plot for the spillover region model using a Gaussian distribution


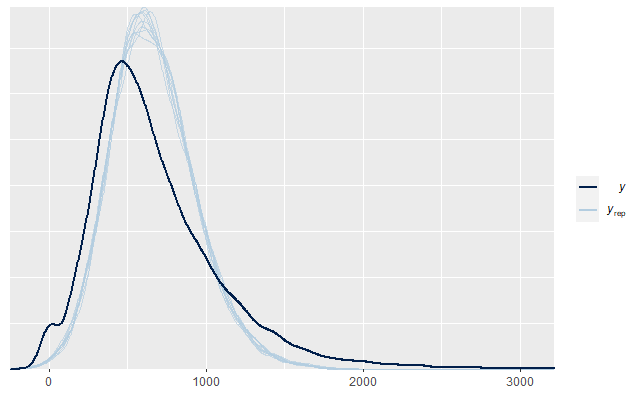


**Figure S2**. The posterior predictive plot for the spillover region model using a hurdle lognormal distribution


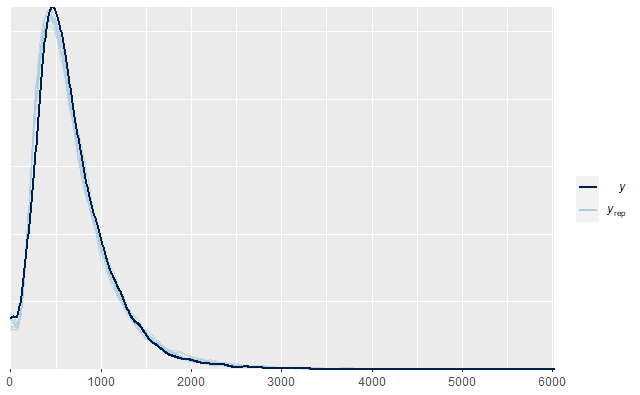


# Final models

## Basic model for pronoun

|  | **Estimate** | **Est.Error** | **l-95% CI** | **u-95% CI** | **Rhat** | **Bulk_ESS** | **Tail_ESS** |
| --- | --- | --- | --- | --- | --- | --- | --- |
| Intercept | 5.0591584 | 0.0210627 | 5.0178238 | 5.0999960 | 1.0002892 | 10567 | 18016 |
| Hu_Intercept | -1.3178290 | 0.0950488 | -1.5087973 | -1.1370524 | 1.0008179 | 10764 | 18367 |
| Noun type | -0.0038538 | 0.0064420 | -0.0163712 | 0.0088963 | 1.0000954 | 43571 | 28182 |
| Pronoun | 0.0034696 | 0.0093201 | -0.0149562 | 0.0216807 | 1.0001040 | 26214 | 26408 |
| Noun, gendered vs genderless | -0.0037693 | 0.0065505 | -0.0166031 | 0.0090824 | 1.0000116 | 44637 | 26466 |
| Experience with hen | 0.0162709 | 0.0313610 | -0.0461803 | 0.0784006 | 1.0000472 | 17615 | 22369 |
| Attitudes towards hen | 0.0267573 | 0.0385218 | -0.0481651 | 0.1021600 | 1.0001841 | 11807 | 18466 |
| Trial | -0.0066653 | 0.0119830 | -0.0302916 | 0.0170800 | 1.0002284 | 32530 | 25964 |
| Light level | 0.0160522 | 0.0168148 | -0.0169550 | 0.0490146 | 1.0004126 | 10408 | 18771 |
| Noun type:Pronoun | 0.0004532 | 0.0066323 | -0.0125661 | 0.0134693 | 1.0000256 | 43519 | 28177 |
| Pronoun:Noun gender | 0.0002680 | 0.0066914 | -0.0128265 | 0.0133444 | 1.0000784 | 40915 | 29115 |
| Pronoun:Experience with hen | -0.0027674 | 0.0153146 | -0.0325875 | 0.0282462 | 0.9999876 | 26028 | 22950 |
| Pronoun:Attitudes  towards hen | -0.0100075 | 0.0181951 | -0.0451149 | 0.0257530 | 1.0003576 | 22942 | 25937 |
| Pronoun:Trial | -0.0008160 | 0.0111036 | -0.0225420 | 0.0209841 | 1.0003070 | 39439 | 27735 |
| Pronoun:Light level | -0.0019993 | 0.0074632 | -0.0165757 | 0.0125653 | 1.0000153 | 34975 | 29165 |
| Hu_Noun type | -0.0025607 | 0.0385462 | -0.0783286 | 0.0732588 | 1.0000350 | 31569 | 25388 |
| Hu_Pronoun | -0.0403855 | 0.0369732 | -0.1128857 | 0.0335758 | 1.0000900 | 32515 | 26309 |
| Hu_Noun gender | 0.0270647 | 0.0395436 | -0.0503695 | 0.1053287 | 1.0000070 | 33082 | 28103 |
| Hu_Experience with hen | 0.0356582 | 0.0848811 | -0.1316426 | 0.2011774 | 1.0001407 | 32016 | 27836 |
| Hu_Trial | -0.0542120 | 0.0595881 | -0.1707718 | 0.0626730 | 1.0002373 | 31490 | 27662 |
| Hu_Light level | 0.0472643 | 0.0705301 | -0.0923455 | 0.1846836 | 1.0000209 | 21788 | 24870 |

## Model including background variables for pronoun

|  | **Estimate** | **Est.Error** | **l-95% CI** | **u-95% CI** | **Rhat** | **Bulk_ESS** | **Tail_ESS** |
| --- | --- | --- | --- | --- | --- | --- | --- |
| Intercept | 5.0317451 | 0.0199777 | 4.9925998 | 5.0711276 | 1.0001413 | 25688 | 26239 |
| Hu_Intercept | -1.2439506 | 0.0707382 | -1.3844349 | -1.1059967 | 0.9999695 | 20836 | 26662 |
| Noun type | -0.0039080 | 0.0064589 | -0.0165510 | 0.0087057 | 1.0001669 | 67850 | 28391 |
| Pronoun | 0.0052184 | 0.0101008 | -0.0145852 | 0.0250166 | 0.9999728 | 30981 | 29005 |
| Noun, gendered vs genderless | -0.0038805 | 0.0065139 | -0.0167764 | 0.0088756 | 1.0002961 | 63684 | 27437 |
| Experience with hen | 0.0232081 | 0.0307788 | -0.0374530 | 0.0833859 | 1.0002565 | 21468 | 26289 |
| Attitudes towards hen | 0.1066795 | 0.0394789 | 0.0292880 | 0.1840272 | 1.0002480 | 23972 | 27005 |
| Trial | -0.0067685 | 0.0119775 | -0.0303137 | 0.0169030 | 1.0000996 | 49282 | 28594 |
| Light level | 0.0277559 | 0.0160099 | -0.0034950 | 0.0593410 | 1.0002134 | 23868 | 25370 |
| Age | -0.0469018 | 0.0582335 | -0.1595154 | 0.0676835 | 1.0000552 | 33476 | 29688 |
| Gender | 0.0059642 | 0.0351831 | -0.0634894 | 0.0754972 | 1.0006080 | 21917 | 26058 |
| Identifying as feminist | -0.0628949 | 0.0169180 | -0.0963642 | -0.0297522 | 1.0002296 | 26693 | 26786 |
| Noun type:Pronoun | 0.0004623 | 0.0066536 | -0.0126484 | 0.0135213 | 1.0001980 | 64540 | 29828 |
| Pronoun:Noun gender | 0.0001684 | 0.0066871 | -0.0129178 | 0.0132424 | 1.0004262 | 56925 | 28768 |
| Pronoun:Experience with hen | -0.0044861 | 0.0155342 | -0.0344401 | 0.0268791 | 1.0001267 | 32806 | 27468 |
| Pronoun:Attitudes towards hen | -0.0150584 | 0.0212276 | -0.0565965 | 0.0265262 | 1.0000491 | 25200 | 28419 |
| Pronoun:Trial | -0.0009322 | 0.0112387 | -0.0229299 | 0.0211864 | 0.9999740 | 58159 | 29072 |
| Pronoun:Light level | -0.0020669 | 0.0079591 | -0.0177553 | 0.0134020 | 1.0000722 | 46200 | 28436 |
| Pronoun:Age | -0.0056191 | 0.0336674 | -0.0711540 | 0.0607543 | 1.0000228 | 45689 | 31404 |
| Pronoun:Gender | -0.0022499 | 0.0183489 | -0.0385285 | 0.0334041 | 1.0001001 | 43231 | 30340 |
| Pronoun:Identifying as feminist | 0.0016930 | 0.0082332 | -0.0142692 | 0.0181750 | 1.0001721 | 38043 | 27369 |
| Hu_Noun type | -0.0055631 | 0.0346984 | -0.0742817 | 0.0626045 | 1.0002725 | 59623 | 27878 |
| Hu_Pronoun | -0.0426885 | 0.0341308 | -0.1094899 | 0.0245005 | 1.0000116 | 59962 | 28692 |
| Hu_Noun gender | 0.0227109 | 0.0352343 | -0.0457089 | 0.0921744 | 0.9999899 | 57430 | 29384 |
| Hu_Experience with hen | 0.0518180 | 0.0757610 | -0.0977999 | 0.1981236 | 1.0000071 | 36745 | 29741 |
| Hu_Trial | -0.0615172 | 0.0508816 | -0.1608862 | 0.0389897 | 1.0001766 | 61363 | 27296 |
| Hu_Light level | 0.0534006 | 0.0593137 | -0.0629832 | 0.1690743 | 1.0003416 | 27770 | 27786 |
| Hu_Age | 0.0222945 | 0.0946702 | -0.1652497 | 0.2070878 | 1.0000893 | 60826 | 27725 |
| Hu_Gender | 0.0178773 | 0.0835402 | -0.1454232 | 0.1800970 | 1.0001780 | 41810 | 29133 |

## Basic model for pronoun spillover

| **Estimate** | **Est.Error** | **l-95% CI** | **u-95% CI** | **Rhat** | **Bulk_ESS** | **Tail_ESS** |  |
| --- | --- | --- | --- | --- | --- | --- | --- |
| Intercept | 6.3985964 | 0.0468531 | 6.3055838 | 6.4902972 | 1.0007634 | 3547 | 7257 |
| Hu_Intercept | -4.3401411 | 0.1400046 | -4.6241248 | -4.0747086 | 0.9999846 | 41480 | 27954 |
| Noun type | -0.0039923 | 0.0081342 | -0.0198468 | 0.0121027 | 1.0001291 | 32311 | 28330 |
| Pronoun | 0.0398071 | 0.0102128 | 0.0197109 | 0.0597100 | 1.0000345 | 28775 | 27842 |
| Noun, gendered vs genderless | 0.0007981 | 0.0069034 | -0.0128271 | 0.0142339 | 1.0002846 | 51101 | 27826 |
| Noun, feminine vs masculine | -0.0064712 | 0.1202455 | -0.2422036 | 0.2284020 | 1.0003109 | 4853 | 8149 |
| Experience with hen | 0.0857497 | 0.0438591 | -0.0015944 | 0.1712852 | 1.0005271 | 13366 | 20604 |
| Attitudes towards hen | -0.0211494 | 0.0588440 | -0.1357486 | 0.0944836 | 1.0001225 | 10994 | 18757 |
| Trial | -0.0843851 | 0.0185411 | -0.1206225 | -0.0480077 | 1.0000978 | 19673 | 25417 |
| Light level | -0.0119701 | 0.0251761 | -0.0616503 | 0.0373411 | 1.0002941 | 10237 | 18453 |
| Noun type:Pronoun | 0.0156034 | 0.0081222 | -0.0003921 | 0.0316071 | 1.0003337 | 25971 | 26441 |
| Pronoun:Noun gender | -0.0257972 | 0.0067709 | -0.0390480 | -0.0125185 | 0.9999744 | 52916 | 27379 |
| Pronoun:Experience with hen | 0.0008172 | 0.0159650 | -0.0303308 | 0.0323550 | 1.0000228 | 28415 | 26754 |
| Pronoun:Attitudes towards hen | -0.0071275 | 0.0194590 | -0.0456133 | 0.0314138 | 1.0001904 | 27368 | 27762 |
| Pronoun:Trial | -0.0155535 | 0.0121556 | -0.0396541 | 0.0083394 | 1.0001044 | 40836 | 29217 |
| Pronoun:Light level | -0.0058509 | 0.0080746 | -0.0217698 | 0.0101582 | 1.0000863 | 35409 | 28405 |
| Hu_Noun type | -0.0608966 | 0.0694861 | -0.1982477 | 0.0748133 | 1.0002675 | 58610 | 25561 |
| Hu_Pronoun | -0.0403516 | 0.0706392 | -0.1793304 | 0.0985181 | 1.0000754 | 57249 | 26281 |
| Hu_Noun gender | -0.0419345 | 0.0711996 | -0.1819065 | 0.0972095 | 1.0003209 | 50838 | 26802 |
| Hu_Experience with hen | -0.0076482 | 0.0909875 | -0.1857871 | 0.1693514 | 1.0002609 | 53168 | 27027 |
| Hu_Trial | 0.0090342 | 0.0873583 | -0.1610652 | 0.1804607 | 1.0000823 | 56576 | 27954 |
| Hu_Light level | 0.1057307 | 0.0826818 | -0.0581793 | 0.2680945 | 0.9999937 | 48905 | 28812 |

## Model including background variables for pronoun spillover

| **Estimate** | **Est.Error** | **l-95% CI** | **u-95% CI** | **Rhat** | **Bulk_ESS** | **Tail_ESS** |  |
| --- | --- | --- | --- | --- | --- | --- | --- |
| Intercept | 6.3632219 | 0.0467299 | 6.2703617 | 6.4539662 | 1.0012690 | 3533 | 8923 |
| Hu_Intercept | -4.3408447 | 0.1403845 | -4.6250657 | -4.0755057 | 1.0000552 | 35503 | 25346 |
| Noun type | -0.0041542 | 0.0080754 | -0.0201615 | 0.0117278 | 1.0002114 | 31398 | 24814 |
| Pronoun | 0.0373461 | 0.0113887 | 0.0149776 | 0.0595342 | 1.0000992 | 24111 | 23852 |
| Noun, gendered vs genderless | 0.0009101 | 0.0068330 | -0.0125848 | 0.0143514 | 1.0002669 | 47514 | 26001 |
| Noun, feminine vs masculine | -0.0064712 | 0.1202455 | -0.2422036 | 0.2284020 | 1.0003109 | 4853 | 8149 |
| Experience with hen | 0.0832614 | 0.0403723 | 0.0043271 | 0.1633878 | 1.0001008 | 20626 | 21804 |
| Attitudes towards hen | 0.0986153 | 0.0636383 | -0.0263758 | 0.2235019 | 1.0001134 | 16742 | 23377 |
| Trial | -0.0840762 | 0.0186752 | -0.1206735 | -0.0470660 | 1.0001349 | 19269 | 22997 |
| Light level | 0.0087177 | 0.0244517 | -0.0395404 | 0.0565984 | 1.0002834 | 15455 | 21725 |
| Age | 0.0007937 | 0.1024630 | -0.2037946 | 0.2022085 | 1.0003440 | 22081 | 22104 |
| Gender | 0.0182848 | 0.0581484 | -0.0954819 | 0.1318558 | 1.0001050 | 21053 | 23573 |
| Identifying as feminist | -0.1030606 | 0.0251941 | -0.1523306 | -0.0528284 | 1.0002428 | 17021 | 21907 |
| Noun type:Pronoun | 0.0157628 | 0.0081236 | -0.0002721 | 0.0316077 | 1.0000524 | 26174 | 24059 |
| Pronoun:Noun gendered vs genderless | -0.0256769 | 0.0066990 | -0.0388711 | -0.0126356 | 1.0002338 | 47156 | 25479 |
| Pronoun: Noun, feminine vs masculine | -0.0124484 | 0.1202556 | -0.2236280 | 0.2501365 | 1.0002956 | 4901 | 8132 |
| Pronoun:Experience with hen | 0.0009159 | 0.0171507 | -0.0329444 | 0.0349445 | 1.0001630 | 26175 | 24292 |
| Pronoun:Attitudes towards hen | 0.0002689 | 0.0228747 | -0.0442752 | 0.0455634 | 1.0001484 | 21412 | 25096 |
| Pronoun:Trial | -0.0156653 | 0.0120627 | -0.0395266 | 0.0081189 | 1.0000696 | 41961 | 26335 |
| Pronoun:Light level | -0.0051908 | 0.0086753 | -0.0221120 | 0.0118335 | 1.0003065 | 32957 | 25839 |
| Pronoun:Age | 0.0095518 | 0.0387342 | -0.0661998 | 0.0859672 | 1.0000359 | 29854 | 25236 |
| Pronoun:Gender | -0.0117673 | 0.0201654 | -0.0510564 | 0.0277989 | 0.9999274 | 30416 | 26715 |
| Pronoun:Identifying as feminist | -0.0107845 | 0.0090614 | -0.0285422 | 0.0072135 | 1.0000823 | 25348 | 25385 |
| Hu_Noun type | -0.0606292 | 0.0692567 | -0.1970707 | 0.0756871 | 1.0001590 | 53749 | 23603 |
| Hu_Pronoun | -0.0402825 | 0.0702481 | -0.1779035 | 0.0974854 | 1.0005208 | 51820 | 23243 |
| Hu_Noun gender | -0.0416557 | 0.0703080 | -0.1800702 | 0.0943597 | 1.0000742 | 54067 | 24522 |
| Hu_Experience with hen | -0.0073009 | 0.0907512 | -0.1864466 | 0.1701628 | 1.0000255 | 54176 | 23790 |
| Hu_Trial | 0.0092919 | 0.0867445 | -0.1598479 | 0.1788465 | 1.0003062 | 53174 | 23002 |
| Hu_Light level | 0.1056434 | 0.0823914 | -0.0568984 | 0.2666328 | 1.0000280 | 40631 | 24417 |
| Hu_Age | 0.0037724 | 0.0992310 | -0.1891993 | 0.1985141 | 1.0009286 | 53589 | 22060 |
| Hu_Gender | -0.0109842 | 0.0940317 | -0.1965574 | 0.1731940 | 0.9999946 | 60099 | 22661 |

## Basic model for noun

| **Estimate** | **Est.Error** | **l-95% CI** | **u-95% CI** | **Rhat** | **Bulk_ESS** | **Tail_ESS** |  |
| --- | --- | --- | --- | --- | --- | --- | --- |
| Intercept | 5.9735523 | 0.0420521 | 5.8901036 | 6.0559391 | 1.0000706 | 14164 | 21411 |
| Hu_Intercept | -1.3457193 | 0.0774824 | -1.5003496 | -1.1957496 | 1.0004264 | 14279 | 21138 |
| Noun type | -0.0067452 | 0.0122390 | -0.0310422 | 0.0171774 | 0.9999852 | 49322 | 28383 |
| Pronoun | -0.0100258 | 0.0177434 | -0.0448178 | 0.0249256 | 0.9999968 | 33148 | 29137 |
| Noun, gendered vs genderless | 0.0002345 | 0.0123618 | -0.0238375 | 0.0244281 | 1.0000377 | 52999 | 28370 |
| Experience with hen | 0.0322792 | 0.0552630 | -0.0765066 | 0.1397496 | 0.9999642 | 24963 | 25652 |
| Attitudes towards hen | -0.0301753 | 0.0670101 | -0.1613709 | 0.1001262 | 1.0002270 | 20839 | 25123 |
| Trial | -0.0340400 | 0.0206412 | -0.0747263 | 0.0069183 | 1.0001705 | 47179 | 27107 |
| Light level | 0.0119924 | 0.0346135 | -0.0564680 | 0.0792672 | 1.0001337 | 16953 | 22782 |
| Noun type:Pronoun | 0.0115748 | 0.0121246 | -0.0120949 | 0.0352383 | 1.0000191 | 49645 | 27676 |
| Pronoun:Noun gender | -0.0076625 | 0.0125980 | -0.0324835 | 0.0171536 | 1.0000730 | 50506 | 28117 |
| Pronoun:Experience with hen | -0.0116852 | 0.0289627 | -0.0679413 | 0.0466340 | 1.0001451 | 32154 | 26240 |
| Pronoun:Attitudes towards hen | -0.0269509 | 0.0335390 | -0.0926329 | 0.0390206 | 1.0000198 | 33189 | 28628 |
| Pronoun:Trial | -0.0063793 | 0.0202923 | -0.0464328 | 0.0334030 | 1.0003454 | 48272 | 28867 |
| Pronoun:Light level | 0.0094347 | 0.0147214 | -0.0190268 | 0.0384699 | 1.0000354 | 35984 | 28346 |
| Hu_Noun type | 0.0192874 | 0.0372970 | -0.0532201 | 0.0934318 | 1.0004243 | 44118 | 28582 |
| Hu_Pronoun | 0.0084160 | 0.0336105 | -0.0581068 | 0.0742252 | 1.0000133 | 58406 | 27167 |
| Hu_Noun gender | -0.0144898 | 0.0355100 | -0.0840002 | 0.0551315 | 1.0000634 | 49046 | 27547 |
| Hu_Experience with hen | 0.0530733 | 0.0768071 | -0.0967264 | 0.2042184 | 1.0000464 | 29900 | 29202 |
| Hu_Trial | 0.0252272 | 0.0509740 | -0.0738959 | 0.1238664 | 1.0001140 | 57442 | 27820 |
| Hu_Light level | 0.0807358 | 0.0606941 | -0.0388477 | 0.1987035 | 1.0000898 | 21244 | 25724 |

## Model including background variables for noun

| **Estimate** | **Est.Error** | **l-95% CI** | **u-95% CI** | **Rhat** | **Bulk_ESS** | **Tail_ESS** |  |
| --- | --- | --- | --- | --- | --- | --- | --- |
| Intercept | 5.9387931 | 0.0410800 | 5.8592865 | 6.0199934 | 1.0000720 | 23744 | 23989 |
| Hu_Intercept | -1.3483419 | 0.0773012 | -1.5015359 | -1.1985379 | 1.0001850 | 14059 | 20286 |
| Noun type | -0.0064028 | 0.0123259 | -0.0305312 | 0.0177044 | 0.9999515 | 51034 | 25013 |
| Pronoun | -0.0036312 | 0.0193461 | -0.0418453 | 0.0340600 | 1.0001770 | 28427 | 26573 |
| Noun, gendered vs genderless | 0.0001980 | 0.0122870 | -0.0239227 | 0.0243552 | 1.0000089 | 48343 | 25033 |
| Experience with hen | 0.0271276 | 0.0533900 | -0.0764586 | 0.1323569 | 0.9999892 | 29146 | 24167 |
| Attitudes towards hen | 0.0526450 | 0.0699345 | -0.0854450 | 0.1886034 | 1.0001027 | 26769 | 23513 |
| Trial | -0.0353859 | 0.0205315 | -0.0751711 | 0.0049488 | 1.0000342 | 48752 | 25638 |
| Light level | 0.0224940 | 0.0340846 | -0.0441544 | 0.0891126 | 1.0000910 | 21644 | 20902 |
| Age | -0.0679126 | 0.0868316 | -0.2370921 | 0.1032428 | 0.9999850 | 44871 | 24337 |
| Gender | 0.0086814 | 0.0667713 | -0.1232240 | 0.1400436 | 0.9999631 | 31241 | 24712 |
| Identifying as feminist | -0.0967092 | 0.0341276 | -0.1626493 | -0.0279722 | 1.0002179 | 22702 | 23348 |
| Noun type:Pronoun | 0.0118160 | 0.0121211 | -0.0119383 | 0.0357455 | 1.0001039 | 49637 | 25150 |
| Pronoun:Noun gender | -0.0080678 | 0.0127066 | -0.0330645 | 0.0166516 | 1.0001023 | 42013 | 25669 |
| Pronoun:Experience with hen | -0.0102356 | 0.0298936 | -0.0683570 | 0.0496821 | 1.0000322 | 28363 | 24786 |
| Pronoun:Attitudes towards hen | -0.0468388 | 0.0383099 | -0.1217109 | 0.0287167 | 1.0001302 | 26353 | 25799 |
| Pronoun:Trial | -0.0064383 | 0.0198929 | -0.0448462 | 0.0328290 | 0.9999661 | 48307 | 26390 |
| Pronoun:Light level | 0.0008731 | 0.0153666 | -0.0290522 | 0.0311355 | 1.0000456 | 31339 | 26177 |
| Pronoun:Age | 0.0293303 | 0.0608654 | -0.0915347 | 0.1472640 | 0.9999902 | 34054 | 27275 |
| Pronoun:Gender | -0.0234426 | 0.0357382 | -0.0933808 | 0.0469747 | 0.9999863 | 34144 | 26083 |
| Pronoun:Identifying as feminist | 0.0108169 | 0.0155795 | -0.0197691 | 0.0410419 | 1.0000712 | 29917 | 25662 |
| Hu_Noun type | 0.0195621 | 0.0374878 | -0.0535555 | 0.0929299 | 0.9999639 | 42374 | 26369 |
| Hu_Pronoun | 0.0083408 | 0.0337610 | -0.0575424 | 0.0749926 | 1.0002384 | 56530 | 25209 |
| Hu_Noun gender | -0.0150536 | 0.0357136 | -0.0859858 | 0.0550715 | 1.0002946 | 50816 | 24743 |
| Hu_Experience with hen | 0.0535176 | 0.0779564 | -0.1003521 | 0.2052313 | 1.0003071 | 26449 | 25099 |
| Hu_Trial | 0.0250869 | 0.0508548 | -0.0742786 | 0.1246190 | 1.0006961 | 55778 | 24106 |
| Hu_Light level | 0.0794805 | 0.0607031 | -0.0394061 | 0.1977908 | 1.0001386 | 20082 | 23591 |
| Hu_Age | 0.0597935 | 0.0964344 | -0.1288593 | 0.2481632 | 0.9999956 | 50825 | 26248 |
| Hu_Gender | 0.0323910 | 0.0857712 | -0.1352403 | 0.2013184 | 1.0000430 | 29355 | 25407 |
